# A pilot study comparing the metabolic profiles of elite-level athletes from different sporting disciplines

**DOI:** 10.1186/s40798-017-0114-z

**Published:** 2018-01-05

**Authors:** Fatima Al-Khelaifi, Ilhame Diboun, Francesco Donati, Francesco Botrè, Mohammed Alsayrafi, Costas Georgakopoulos, Karsten Suhre, Noha A. Yousri, Mohamed A. Elrayess

**Affiliations:** 1 Anti Doping Laboratory Qatar, Sports City, P.O Box 27775, Doha, Qatar; 2 University College London-Medical School, Royal Free Campus, London, NW3 2PF UK; 3 Department of Economics, Mathematics and Statistics, Birkbeck, University of London, London, WC1E 7HX UK; 4 Laboratorio Antidoping, Federazione Medico Sportiva Italiana, Largo Giulio Onesti 1, 00197 Rome, Italy; 5 Department of Physiology and Biophysics, Weill Cornell Medical College in Qatar, Qatar-Foundation, P.O. Box 24144, Doha, Qatar; 6 Department of Genetic Medicine, Weill Cornell Medical College in Qatar, Education City, Qatar-Foundation, P.O. Box 24144, Doha, Qatar; 7 Department of Computer and System Engineering, Alexandria University, Alexandria, Egypt

**Keywords:** Metabolomics, Elite athletes, Power, Endurance, Steroids biosynthesis, Oxidative stress, Energy substrates

## Abstract

**Background:**

The outstanding performance of an elite athlete might be associated with changes in their blood metabolic profile. The aims of this study were to compare the blood metabolic profiles between moderate- and high-power and endurance elite athletes and to identify the potential metabolic pathways underlying these differences.

**Methods:**

Metabolic profiling of serum samples from 191 elite athletes from different sports disciplines (121 high- and 70 moderate-endurance athletes, including 44 high- and 144 moderate-power athletes), who participated in national or international sports events and tested negative for doping abuse at anti-doping laboratories, was performed using non-targeted metabolomics-based mass spectroscopy combined with ultrahigh-performance liquid chromatography. Multivariate analysis was conducted using orthogonal partial least squares discriminant analysis. Differences in metabolic levels between high- and moderate-power and endurance sports were assessed by univariate linear models.

**Results:**

Out of 743 analyzed metabolites, gamma-glutamyl amino acids were significantly reduced in both high-power and high-endurance athletes compared to moderate counterparts, indicating active glutathione cycle. High-endurance athletes exhibited significant increases in the levels of several sex hormone steroids involved in testosterone and progesterone synthesis, but decreases in diacylglycerols and ecosanoids. High-power athletes had increased levels of phospholipids and xanthine metabolites compared to moderate-power counterparts.

**Conclusions:**

This pilot data provides evidence that high-power and high-endurance athletes exhibit a distinct metabolic profile that reflects steroid biosynthesis, fatty acid metabolism, oxidative stress, and energy-related metabolites. Replication studies are warranted to confirm differences in the metabolic profiles associated with athletes’ elite performance in independent data sets, aiming ultimately for deeper understanding of the underlying biochemical processes that could be utilized as biomarkers with potential therapeutic implications.

**Electronic supplementary material:**

The online version of this article (10.1186/s40798-017-0114-z) contains supplementary material, which is available to authorized users.

## Key points

- The emerging data provide a comprehensive snapshot of athletes’ metabolism based on their sports class as well as small molecule markers of fitness, including changes in metabolites reflecting sex steroid hormone biosynthesis and oxidative stress substrates.
- The analysis confirmed previously reported changes in the consumption of energy substrates in glycolysis, lipolysis, adenine nucleotide catabolism, and amino acid catabolism in response to exercise.
- Once replicated and validated, these metabolic signatures could be utilized as biomarkers for excessive trainability associated with elite athletic performance with potential therapeutic implications.

## Background

Excessive training of professional athletes causes alterations in their blood metabolic profile that depends largely on the type and duration of their training regimen [1, 2]. Various behavioral, biochemical, hormonal, and immunological markers are routinely used to assess athletes’ physical status during a training program [3, 4]. Previous studies, however, have demonstrated that conventional tests could not detect the physiological differences between endurance athletes and control subjects, or differences before and after training sensitively [5, 6]. Therefore, a more comprehensive metabolic profiling has been considered in order to identify global physiological changes in response to training.

Metabolomics offers a quantitative measurement of the metabolic profiles associated with exercise in professional athletes in order to identify biomarkers associated with their performance, response to fatigue, and potentially their respective sports-related disorders [5, 7]. Non-targeted metabolomics allows the detection of changes in response to various physiological states such as pre-/post-exercise and offers identification of metabolic signatures with potential translational impact for both professional athletes and general public [8]. These changes include metabolites associated with glucose, lipid, amino acid, and energy metabolism [1, 5], such as those involved in adenosine triphosphate (ATP) synthesis, beta-oxidation of free fatty acids, and ketone bodies [8]. Previous studies in healthy volunteers have demonstrated significantly reduced excretion of amino acids with increased fitness levels and increased fat oxidation rate during exercise [9]. Furthermore, metabolomics profiling of athletes undergoing intensive exercise revealed increase in plasma lactate [10, 11] and adenine breakdown products [12], indicating anaerobic metabolism and ATP cycling, respectively. Further studies of the effect of exercise showed elevated tricarboxylic acid (TCA) cycle intermediates, markers of aerobic energy production, in skeletal muscle biopsies [13, 14]. Intensive exercise was also shown to trigger changes in the levels of amino acids, including a moderate uptake of glutamate in skeletal muscle leading to release of alanine to promote ammonia metabolism [11, 15, 16], with corresponding changes in plasma concentrations of these metabolites [17, 18]. Elevation in serum levels of sex steroid hormones was also reported in endurance athletes only in response to high exercise intensities [19].

Athletes who have competed in national or international sports events are considered elite athletes and have been classified into two broad types according to the kind and intensity of exercise: dynamic (isotonic) and static (isometric) [20, 21]. The dynamic exercise represents changes in the muscle length due to regular contractions producing a limited intramuscular power. These changes are characteristic of high-endurance sports such as marathon running, cycling, or long-distance triathlons. Static exercise, on the other hand, leads to a greater intramuscular power with little changes in muscle length and is characteristic to power sporting events such as sprinting, jumping, throwing, and weightlifting. Some sports, however, require both power and endurance such as boxing and rowing. Dynamic exercise can also be further characterized based on the maximal oxygen uptake percentage (VO_2_) achieved with maximum cardiac output. Static exercise can too be sub-categorized in relation to maximal voluntary contraction percentage (MVC) gained with increasing blood pressure [21].

Despite multiple studies focusing on the impact of exercise on athletes’ metabolomics profiling, the metabolic differences between high- and moderate-power and endurance athletes remain to be explored. This study aims to identify the metabolic signature that differentiates high- and moderate-power and endurance elite athletes and to identify the potential metabolic pathways that underlie these differences. Assessment of these changes could provide valuable measures of the current physical status of the athletes and their adaptation to training, which may help directing future training programs, preventing potential disorders associated with excessive exercise as well as improving their overall performance.

## Methods

### Study design

Study participants included in this study were 191 consented elite athletes (171 males and 20 females) from different sports disciplines who participated in national or international sports events and tested negative for doping substances at anti-doping laboratories in Qatar and Italy. Spare serum samples collected for anti-doping human growth hormone tests were used for metabolomics studies. Briefly, samples were either collected IN or OUT of competition. Once collected, samples were delivered to the anti-doping labs within 36 h under cooling conditions. Once received, samples were immediately centrifuged to separate the serum and then stored at − 20 °C until analysis. Only information related to type of sport and athlete’s gender were available to researches. All other information was not available, including age, ethnicity, or the time of recruitment (pre- or post-exercise), due to the strict anonymization process undertaken by anti-doping laboratories and those dictated by study’s ethics. This study was performed in line with the World Medical Association Declaration of Helsinki. All protocols were approved by the Institutional Research Board of anti-doping lab Qatar (F2014000009). Sport types can be dichotomized into low, moderate, and high dynamic or static groups based on associated peak dynamic (VO_2_) and peak static (MVC) components achieved during competition, as suggested previously [21]. In our study, few athletes belonged to low levels of endurance and power, therefore were merged with the corresponding moderate class of endurance and power, respectively (Table 1A). For statistical analysis, endurance and power athletes were each represented by a categorical variable with two levels (high and moderate, Table 1B). Table 1 further lists the number of participants per sport type in each class and their genders.

**Table 1:**
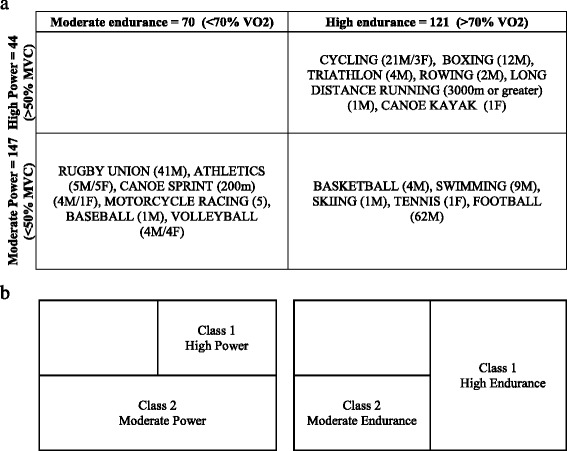
Classification of study participants

(A) Distribution of elite athletes in various categories based on sport type-associated peak dynamic (maximal oxygen uptake percentage; VO_2_) and peak static (maximal voluntary muscle contraction percentage; MVC) components achieved during competition as described previously [21]. The number and gender (M for males and F for females) of participants in each group are also indicated. (B) Categorization of sport types into classes based on power alone regardless of endurance (left) and similarly for endurance alone ignoring power (right); these classes were used in the statistical analysis

### Metabolomics

Metabolomics profiling was performed using established protocols at Metabolon, Durham, NC, USA. All methods utilized a Waters ACQUITY ultra-performance liquid chromatography (UPLC) and a Thermo Scientific Q-Exactive high resolution/accurate mass spectrometer interfaced with a heated electrospray ionization (HESI-II) source and Orbitrap mass analyzer operated at 35,000 mass resolution. The detailed description of the liquid chromatography-mass spectrometry (LC-MS) methodology was previously described [22] and is summarized in the Additional file 1. Briefly, serum samples were methanol extracted to remove the protein fraction. The resulting extract was divided into five fractions: two for analysis by two separate reverse phase (RP)/UPLC-MS/MS methods with positive ion mode electrospray ionization (ESI), one for analysis by RP/UPLC-MS/MS with negative ion mode ESI, one for analysis by hydrophilic interaction chromatography (HILIC)/UPLC-MS/MS with negative ion mode ESI, and one sample was reserved for backup. Raw data was extracted, peak-identified, and quality control-processed using Metabolon’s hardware and software [23]. Compounds were identified by comparison to library entries of purified standards or recurrent unknown entities with more than 3300 commercially available purified standard compounds. Library matches for each compound were checked for each sample and corrected if necessary [22]. Asterisks (*) indicated on IDs of some metabolites in Tables 2 and 3, Additional file 2: Tables S2–S3 and S5–S8 refer to compounds that have not been officially confirmed based on a standard, but their identities are known with confidence.

**Table 2:** Metabolites differentiating between moderate- and high-endurance athletes (Bonferroni significance)

Metabolite	Sub-pathway	Fold change	Bonferroni *p* value
1-stearoyl-GPC (18:0)	Lysolipid	− 0.15595	1.72E-05
Vanillylmandelate (VMA)	Phenylalanine and tyrosine metabolism	0.415133	2.29E-05
21-hydroxypregnenolone disulfate	Steroid	0.365863	0.000107398
Palmitoyl-linoleoyl-glycerol (16:0/18:2) [2]*	Diacylglycerol	− 0.46764	0.000130998
Tartronate (hydroxymalonate)		0.290077	0.000657114
Palmitoyl-linoleoyl-glycerol (16:0/18:2) [1]*	Diacylglycerol	− 0.42202	0.00090176
1-palmitoleoyl-GPC (16:1)*	Lysolipid	− 0.22642	0.001172265
Cortisone	Steroid	0.395892	0.001489996
Citrate	TCA cycle	0.200056	0.001784274
Succinimide	Polyamine metabolism	0.279317	0.002636335
Stearoylcarnitine (C18)	Fatty acid metabolism (acyl carnitine)	− 0.28394	0.002953686
Trans-4-hydroxyproline	Urea cycle; arginine and proline metabolism	− 0.27783	0.00295413
4-guanidinobutanoate	Polyamine metabolism	− 0.44969	0.003796483
Dihomo-linoleoylcarnitine (C20:2)*	Fatty acid metabolism (acyl carnitine)	−0.33166	0.005028391
1-(1-enyl-palmitoyl)-2-oleoyl-GPC (P-16:0/18:1)*	Plasmalogen	0.145176	0.005178692
1-palmitoyl-GPC (16:0)	Lysolipid	− 0.11595	0.005429078
Linoleoyl-linoleoyl-glycerol (18:2/18:2) [1]*	Diacylglycerol	− 0.54301	0.005827373
Gamma-glutamylglutamate	Gamma-glutamyl amino acid	− 0.42069	0.006242208
Pregnanediol-3-glucuronide	Steroid	0.44061	0.006441558
Palmitoyl-arachidonoyl-glycerol (16:0/20:4) [2]*	Diacylglycerol	− 0.47247	0.008366458
1-palmitoyl-2-stearoyl-GPC (16:0/18:0)	Phospholipid metabolism	− 0.1648	0.009358338
Cortisol	Steroid	0.471022	0.009967366
Linoleoyl-linolenoyl-glycerol (18:2/18:3) [2]*	Diacylglycerol	− 0.53635	0.012030273
Homoarginine	Urea cycle; arginine and proline metabolism	− 0.22816	0.013313047
Palmitoleoyl-linoleoyl-glycerol (16:1/18:2) [1]*	Diacylglycerol	− 0.42989	0.015554355
Lactosyl-N-palmitoyl-sphingosine (d18:1/16:0)	Sphingolipid metabolism	0.131658	0.017917489
3-hydroxydecanoate	Fatty acid, monohydroxy	0.346756	0.018411909
Pregnenolone sulfate	Steroid	0.332031	0.01854452
Pregnenolone steroid monosulfate*	Steroid	0.292548	0.024089561
Leukotriene B4	Eicosanoid	− 0.84063	0.027085708
Vanillactate	Phenylalanine and tyrosine metabolism	0.214757	0.028124765
12-HETE	Eicosanoid	− 0.63302	0.028449419
Acetylcarnitine (C2)	Fatty acid metabolism (acyl carnitine)	0.337317	0.033107027
N1-methyladenosine	Purine metabolism, adenine containing	0.121048	0.036870759
Isovalerate	Leucine, isoleucine and valine metabolism	− 0.52129	0.039358891
5-hydroxylysine	Lysine metabolism	− 0.39575	0.040606024
1,3,7-trimethylurate	Xanthine metabolism	0.671617	0.045828468
Fructose	Fructose, mannose and galactose metabolism	0.391699	0.053677595

Asterisks (*) indicated on IDs of some metabolites refer to compounds that have not been officially confirmed based on a standard, but their identities are known with confidence

**Table 3:** Metabolites that differentiate moderate- versus high-power athletes

Metabolite	Sub-pathway	Fold change	Bonferroni *p* value
1-palmitoyl-2-palmitoleoyl-GPC (16:0/16:1)*	Phospholipid metabolism	0.577623	5.92E-11
1-palmitoyl-2-oleoyl-GPI (16:0/18:1)*	Phospholipid metabolism	0.42177	1.10E-07
Imidazole lactate	Histidine metabolism	0.447699	1.88E-06
1-stearoyl-2-oleoyl-GPC (18:0/18:1)	Phospholipid metabolism	0.279019	4.51E-06
1-linolenoyl-GPC (18:3)*	Lysolipid	0.414819	1.10E-05
1-linoleoyl-2-linolenoyl-GPC (18:2/18:3)*	Phospholipid metabolism	0.537975	1.11E-05
1-palmitoyl-2-linoleoyl-GPI (16:0/18:2)	Phospholipid metabolism	0.447877	5.88E-05
1-palmitoyl-GPI (16:0)	Lysolipid	0.438221	0.000101
Indolelactate	Tryptophan metabolism	0.30948	0.000178
3-methylxanthine	Xanthine metabolism	0.788924	0.00021
1,2-dilinoleoyl-GPC (18:2/18:2)	Phospholipid metabolism	0.324133	0.000225
1-lignoceroyl-GPC (24:0)	Lysolipid	0.321129	0.000287
1-palmitoyl-2-stearoyl-GPC (16:0/18:0)	Phospholipid metabolism	0.222855	0.000322
N-acetylcarnosine	Dipeptide derivative	− 0.33185	0.000873
1-stearoyl-2-oleoyl-GPI (18:0/18:1)*	Phospholipid metabolism	0.346165	0.001026
N-acetylmethionine	Methionine, cysteine, SAM, and taurine metabolism	− 0.58119	0.001445
1-palmitoyl-2-oleoyl-GPC (16:0/18:1)	Phospholipid metabolism	0.153562	0.002983
Argininate*	Urea cycle; arginine and proline metabolism	0.422405	0.003294
7-methylxanthine	Xanthine metabolism	0.648043	0.004023
Homoarginine	Urea cycle; arginine and proline metabolism	− 0.27429	0.006606
Gamma-glutamylvaline	Gamma-glutamyl amino acid	− 0.3052	0.008009
Sphingosine 1-phosphate	Sphingolipid metabolism	− 0.20846	0.008168
Phenyllactate (PLA)	Phenylalanine and tyrosine metabolism	0.306398	0.009708
Arabitol/xylitol	Pentose metabolism	0.23942	0.015147
1-palmitoleoyl-GPC (16:1)*	Lysolipid	0.229408	0.017685
Methionine sulfone	Methionine, cysteine, SAM, and taurine metabolism	0.308995	0.02004
Guanidinoacetate	Creatine metabolism	− 0.22401	0.035446
1-stearoyl-2-linoleoyl-GPI (18:0/18:2)	Phospholipid metabolism	0.261839	0.036305
Sphingomyelin (d18:2/14:0, d18:1/14:1)*	Sphingolipid metabolism	0.216635	0.036711
4-cholesten-3-one	Sterol	0.242711	0.037246
1-palmitoyl-GPG (16:0)*	Lysolipid	0.309379	0.040079
Cholate	Primary bile acid metabolism	1.182236	0.041373
1-palmitoyl-GPE (16:0)	Lysolipid	0.230631	0.049265
1-stearoyl-2-linoleoyl-GPC (18:0/18:2)*	Phospholipid metabolism	0.118022	0.052877

Asterisks (*) indicated on IDs of some metabolites refer to compounds that have not been officially confirmed based on a standard, but their identities are known with confidence

### Statistical analysis of metabolomics data

#### Multivariate analysis

Metabolomics data were log-transformed to ensure distribution normality. Batch correction was already performed by Metabolon by rescaling each metabolite so that its median is equal to 1. Principle component analysis (PCA) was initially undertaken using multivariate techniques to achieve a global view of the data. PCA components express a linear combination of the metabolites levels weighted by the component’s loading values. Orthogonal partial least square discriminant analysis (OPLS-DA), a supervised multivariate regression technique, was performed to identify components that best differentiate between predefined classes of samples while dissecting orthogonal components which do not differentiate between these classes. In this study, OPLS-DA was used to compare moderate versus high classes of endurance and power separately. Both PCA and OPLS-DA were run using SIMCA 14 with the default metabolite-wise metabolite missingness threshold (percentage of missing metabolite values across the samples) of 50%.

#### Univariate regression and enrichment analysis

Linear models for association analysis were run using the R statistical package (version 2.14, www.r-project.org/). A model incorporating power and endurance as a categorical variable with two levels (moderate and high) was used. Incorporating both endurance and power in the same model made it possible to examine the effect of power while correcting for endurance and vice versa. This is sensible because the high-endurance class features a mixture of high- and moderate-power sports while the moderate-endurance class features only moderate-power sports. An opposite pattern is observed with power (Table 1B). With both analyses, covariates including gender, hemolysis levels (determined visually by Metabolon), and PCA components 1 and 2 were included in the model. A stringent Bonferroni level of significance of *p* ≤ 0.05/743 = 6.72 × 10 − 5 was used to infer association. False discovery rate (FDR) multiple testing correction was also performed. All *p* values included in Tables 2 and 3, Additional file 2: Tables S2–S6 are reported after performing the described multiple testing correction. In order to identify metabolites that were associated with endurance or power differently in males versus females (endurance/power × gender), an interaction term was added to the model. For simplicity, when conducting the interaction analysis, both endurance and power were used as continuous variables (since both come in only two levels); hence, the analysis was reduced to testing differences in the beta values between males and females (where beta expressed the slope measuring the effect of either power or endurance).

Function enrichment analysis was performed using the one-tailed Wilcoxon sum of the ranks test. For a given biological function, the test assesses the probability of observing the identified ranks of related metabolites from the linear model analysis by chance. To gain further insight into the biochemistry of identified metabolites, the Kyoto Encyclopedia of Genes and Genomes (KEGG) pathways were utilized. For heatmap analysis, metabolites were *z*-scaled by subtracting their means followed by division by standard deviations.

## Results

### Multivariate analysis of athlete metabolomics data

**Fig. 1:**
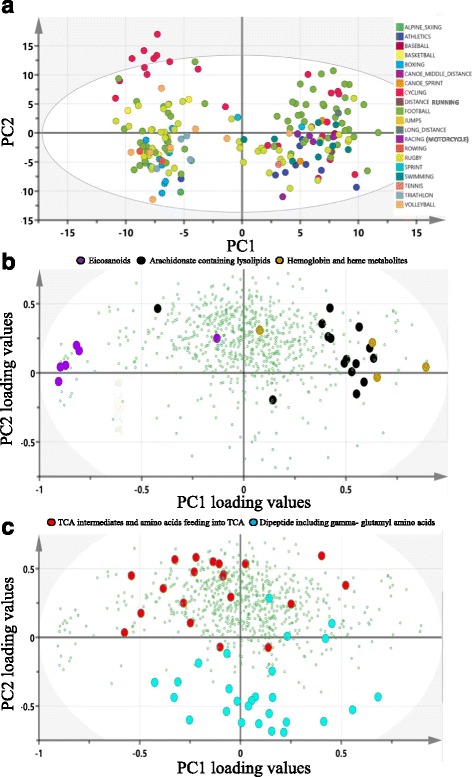
PCA analysis of athlete metabolomics data. **a** A score plot of PC1 and PC2 indicating clustering of samples into two groups according to PC1. Neither PCs is explained by sport type or class. **b**, **c** Loading plots offering clues on what the two PCs may represent: The heme/hemoglobin metabolites suggests a hemolysis signature for PC1 (**b**) while the TCA energy metabolite highlighted by PC2 indicates an energy generating process which may be associated with exercise (**c**)

Non-targeted metabolomics was applied to determine the metabolic signatures of 191 elite athletes. PCA components 1 and 2 (PC1 and PC2) captured together 25% of the variance in the data. PC1 revealed two clusters of samples, which were not explained by gender, sport types, or classes (Fig. 1a). Examination of the loading plot in Fig. 1b revealed a concentration of hemoglobin and heme metabolites at the positive end of PC1. Furthermore, a *t* test comparing the hemolysis measurement, between the two clusters of samples revealed by PC1, was significant at the 0.01 significance level. These results led to the conclusion that PC1 captured the extent of hemolysis in the samples. Interestingly, there was also an enrichment of arachidonate phospholipid metabolites at the positive end of PC1 as oppose to an enrichment of eicosanoids at the negative end. While the biochemical link between the two sets of metabolites is an obvious substrate/product relationship, the link to hemolysis was rather obscure. There were no clusters of samples according to PC2 (Fig. 1a). A closer look at the loading plot revealed that TCA energy metabolites and amino acids that feed into TCA cycle were mostly located at the positive end of PC2 (Fig. 1c). Moreover, a significant positive correlation between previously identified changes in metabolites following 1 hour post-endurance exercise [1], also listed in Additional file 2: Table S1, and our PC2 loading values for the same metabolites (*R* = 0.6, *p* = 0.005) was identified. The enrichment of dipeptides at the negative end of PC2 could indicate an opposing anabolic effect. Although PCA did not explain sport classes, it provided clues of possible confounders (hemolysis and pre/post exercise) that we corrected for subsequent analyses.

**Fig. 2:**
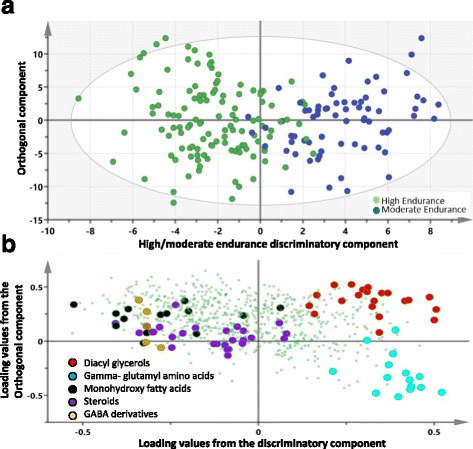
OPLS-DA model comparing moderate- versus high-endurance classes of elite athletes. **a** A score plot showing the class-discriminatory component (*x*-axis) versus orthogonal component (*y*-axis). **b** The corresponding loading plot showing a clustering of steroids and monohydroxy-fatty acids at the high end of endurance opposed by a clustering of diacyl-glycerols and gamma-glutamyl amino acids at the negative end

Unlike PCA, OPLS-DA can identify sets of metabolites that best distinguish between predefined classes of samples. An OPLS-DA analysis comparing moderate versus high classes of endurance revealed one class-discriminatory component accounting for 66.7% of the variation in the data due to endurance level (R-squared-Y = 0.66, Q-squared = 0.45) (Fig. 2a). The corresponding loading score, shown in Fig. 2b, suggests a reduction in diacyl glycerols and gamma-glutamyl amino acids as oppose to an increase in steroids, GABA derivatives, and monohydroxy fatty acids with higher endurance levels.

**Fig. 3:**
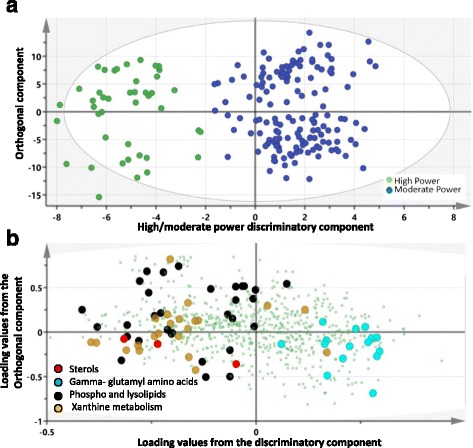
OPLS-DA model of moderate- versus high-power classes of elite athletes. **a** A score plot showing the class-discriminatory component on the *x*-axis versus the first orthogonal component on the *y*-axis. **b** The corresponding loading plot showing a clustering of sterols, lipids, and xanthine metabolites at the high end of power as opposed to enrichment of gamma-glutamyl amino acids at the low end of power

OPLS-DA also revealed a clear separation between moderate versus high power. One significant predictive component explaining 88% of the variation in the power (R-squared-Y = 0.88, Q-squared = 0.52) was identified (Fig. 3a). The loading plot on Fig. 3b suggests a decrease in gamma glutamyl amino acids as oppose to an increase in sterols, phospholipids, lysolipids, and xanthine metabolites with increased power. OPLS results were confirmed by linear model in the following section.

### Univariate association tests and function enrichment analysis

#### Endurance-associated metabolites

A linear model was used to assess the significance of metabolite-associations with the athletes’ class (moderate versus high endurance) after correcting for gender, hemolysis levels, PC1, PC2, and power. Thirty-eight metabolites associated with endurance at a Bonferroni level of significance (*p* ≤ 0.05/743 = 6.72 × 10^−5^) were identified and their associated pathways listed (Table 2). More metabolites associated with endurance at FDR and nominal levels of significance are shown in Additional file 2: Table S2. Similar results were obtained when analysis was restricted to males only (Additional file 2: Table S3).

Enrichment analysis revealed an over-representation of diacylglycerols, gamma-glutamyl amino acids, eicosanoids, and monohydroxy fatty acids (FDR-corrected *p*-value 0.000122, 0.005, 0.017, and 0.04, respectively) among metabolites most strongly associated with endurance, irrespective of the direction of change. The steroid class scored a nominal *p*-value of 0.05 but failed to remain significant after FDR-based multiple testing. Interestingly, these results are in considerable agreement with metabolic effects identified through the OPLS-DA multivariate approach previously discussed (Fig. 2b).

**Fig. 4:**
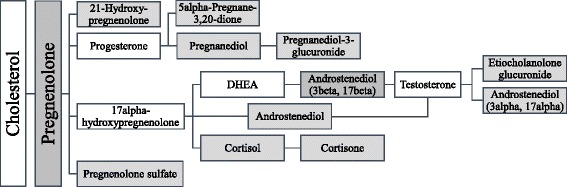
A schematic diagram summarizing the biochemical relationships between steroid metabolites found significantly associated with high endurance (shaded boxes). This is based on the steroid hormone biosynthesis reference pathway (map00140) from the Kyoto Encyclopedia of Genes and Genomes (KEGG)

The results pertaining to steroids are certainly remarkable if replicated and will be elaborated further in the “Discussion” section. It is important to note that in addition to the six Bonferroni significant steroids listed in Table 2, seven more steroid species were FDR significant at alpha = 0.05. These are etiocholanolone glucuronide (FDR *p* value = 0.003); 5alpha-pregnane-3beta,20alpha-diol disulfate (FDR *p* value = 0.01); 5alpha-pregnane-3beta,20beta-diol monosulfate (FDR *p* value = 0.02); androstenediol (3beta,17beta) disulfate (FDR *p* value = 0.025); 5alpha-pregnane-3beta,20alpha-diol monosulfate (FDR *p* value = 0.029); pregnen-dioldisulfate (FDR *p* value = 0.035); and androstenediol (3alpha, 17alpha) monsulfate (FDR *p* value = 0.04). All Bonferroni and FDR significant steroid metabolites were projected onto KEGG Steroid Biosynthesis Pathway to highlight their biochemical inter-relationships (Fig. 4). Significant correlations among the identified steroid metabolites were confirmed (Additional file 3: Fig. S1, Additional file 2: Table S4), suggesting activation of sex steroid biosynthesis pathway in high-endurance athletes.

A part of enrichments of functionally related sets of metabolites, endurance association analysis also revealed individual metabolic effects which are noteworthy. Among these are derivatives of GABA cyclic lactam 2-pyrrolidinone including succinimide (Bonferroni *p* value = 0.00263), acisoga or *N*-(3-acetamidopropyl)pyrrolidin-2-one (FDR *p* value = 0.004), and 2-pyrrolidinone itself (FDR *p* value = 0.03) as well as GABA derivative 4-guanidinobutanoate (Bonferroni *p* value = 0.004). There were significant correlations between 2-pyrrolidinone and its derivatives including succinimide (*R* = 0.15, *p* = 0.04), 4-guanidinobutanoate (*R* = − 0.146, *p* = 0.04), and guanidinosuccinate (*R* = − 0.186, *p* = 0.01), suggesting presence of this drug and its derivatives in high-endurance athletes, also seen in OPL-DA analysis (Fig. 2b).

Other interesting effects include a Bonferroni significant increase in citrate together with an FDR significant increase in 2-methylcitrate (FDR = 0.012). Other associations include acyl carnitines, phospholipids, and sphingolipids among others (Table 2).

### Power associated metabolites

When considering power, the categorical variable “power” becomes the explanatory variable of interest in the previous model and “endurance” becomes a confounder that is corrected for. Thirty-three metabolites were significantly associated with power according to this model; these are listed in (Table 3). Enrichment analysis revealed an over-representation of phospholipids (*p* = 0.00042), lysolipids (*p* = 0.00042), gamma-glutamyl amino acids (*p* = 0.000846), and sterols (*p* = 0.005) amongst metabolites most strongly associated with power. Other significantly changed metabolites in moderate- versus high-power classes included guanidinoacetate, N-acetylcarnosine, cholate, imidazole lactate, indolelactate, and 3-methylxanthine (Table 3).

Among FDR significant changes, an increase in creatine (estimate = 0.6, *p* = 0.001) and a decrease in creatinine (estimate = − 0.1, *p* = 0.002) were also detected in the high-power group although did not reach Bonferroni significance. More metabolites associated with power at FDR level of significance are shown in Additional file 2: Table S5. Similar results were obtained when analysis was restricted to males only (Additional file 2: Table S6).

**Fig. 5:**
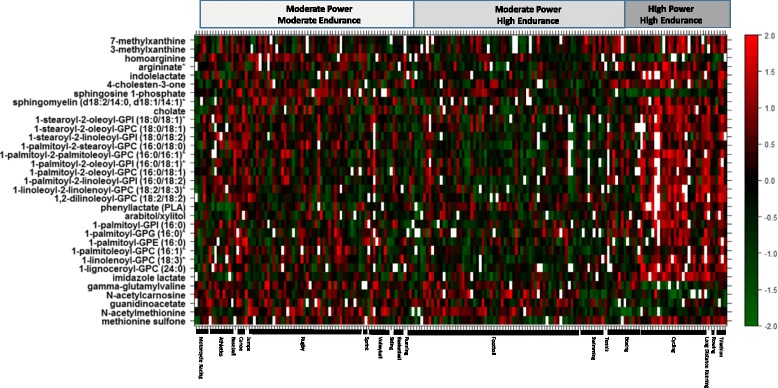
Heatmap of metabolites significantly associated with high endurance from the linear model association analysis (*y*-axis). Samples on *x*-axis were ordered by sports type and group. The color code denotes *z*-scaled values of metabolites after correction of confounders

**Fig. 6:**
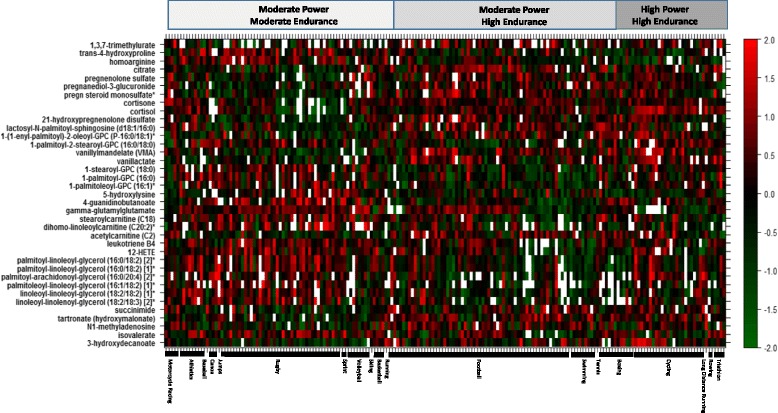
Heatmap of metabolites significantly associated with high power from the linear model association analysis (*y*-axis). Samples on *x*-axis were ordered by sports type and group. The color code denotes *z*-scaled values of metabolites after correction of confounders

Metabolites with FDR corrected *p* values of less than 0.01 from the endurance and power models were projected on the heatmap in Figs. 5 and 6, respectively. The heatmaps give a snapshot summary of the actual intensities of these metabolites after correcting for confounders in the linear model described earlier. Samples were ordered by sports type within their respective sport groups (moderate power/moderate endurance, moderate power/high endurance and high power/high endurance).

### Gender-sports class interaction

Gender-endurance interaction analysis identified 60 significant metabolites with a nominal *p* value (less than 0.05) amongst which none remained significant after FDR correction (Additional file 2: Table S7). As for power, 144 metabolites were differently associated with power between males and females, among which 35 metabolites remained significant after FDR correction (Additional file 2: Table S8).

## Discussion

Metabolic profiling of athletes’ blood in response to exercise has recently revealed unique metabolic signatures associated with various types and durations of exercise [1, 8]. However, metabolomics of elite athletes from different sport disciplines remains to be investigated. In particular, the metabolic pathways of endurance and power athletes should shed light on the molecular mechanisms underlying variations with functional relevance or those that can be used as potential biomarkers for their respective sport class. In this study, metabolomics analysis was utilized to characterize the unique serum metabolic signature of elite athletes who participated in national or international sports events following the successful completion of anti-doping tests. Despite limited information about the participants and possible confounding factors influencing their metabolic profiling, the emerging data revealed significant differences in metabolite levels between high- versus moderate-power and endurance sport types. Inclusion of PC1 and PC2 in the linear model has likely corrected for expected confounders including hemolysis and pre-post exercise effects to reveal common as well as distinctive metabolic mechanisms underlying endurance and power. These include a clear signature of oxidative stress common to both high-power and high-endurance sports alike, yet steroids and polyamine pathways appeared more prominent in endurance, while sterols, adenine-containing purines, and energy metabolites were most evident with power.

### Metabolites associated with endurance

Exercise can cause changes in sex steroid hormone concentrations in the serum of non-athletes as well as athletes [19, 24], including levels of testosterone and cortisol [25, 26]. One interesting finding in this study is the elevated levels of various metabolites involved in sex steroid hormone biosynthesis in the high-endurance athletes. Some of these metabolites were conjugated with one or more sulfate group(s) which renders them inactive. However, these can be reactivated through the activity of enzyme steroid sulfatase [27]. The list of elevated steroids included pregnenolone that mediates biosynthesis of corticosteroids and progesterone and 21-hydroxypregnenolone disulfate that mediates biosynthesis of corticosteroids, corticoids (cortisol and cortisone), various metabolites of progesterone (pregnanediol, 5alpha-pregnane-3beta,20alpha-diol, 5alpha-pregnane-3beta,20beta-diol), testosterone precursor (androstenediol (3beta,17beta)), and testosterone metabolites (etiocholanoloneglucuronide, androstenediol (3alpha, 17alpha)) (Fig. 4). Elevated cortisol-related metabolites in response to sustained aerobic exercise were shown to correlate positively with intensity of exercise as measured by oxygen uptake [28]. However, exercise-induced alterations in sex steroid hormone levels are usually short lived (1–3 h) [19]. The habitual exercise regiments of the elite endurance athletes may have accounted for this maintained systemic increase. Sex steroid hormones play a crucial role in glucose metabolism and protein synthesis in the muscle as well as in the regulation of redox homeostasis [29–31]. Some act as neurosteroids that alter neuronal excitability such as pregnen-dioldisulfate that works as a potent negative allosteric modulator of the GABAA receptor [32] and pregnenolone sulfate that acts as a potent negative allosteric modulator of the GABAA receptor and a weak positive allosteric modulator of the NMDA receptor [33]. The stimulatory effects of steroids on muscle mass, energy generation, and neuronal excitability may have accounted for the higher endurance ability of the high-endurance group compared to their lower endurance counterparts. Given that athletes included in this study have successfully passed anti-doping tests, changes in steroids levels may reflect either enhancement in endogenous anabolic steroids biosynthesis, physiological adaptation to exercise, and/or increased dietary intake. A genetic association study is needed to reveal the potential genetic variants underlying increased activity of enzymes involved in steroid biosynthesis. Interestingly, in addition to elevation in a number of neurosteroids, our data suggested increased elevated levels of a number of GABA derivatives including 2-pyrrolidinone, the cyclic lactate form of GABA [34], its derivatives succinimide, acisoga (N-(3-acetamidopropyl)pyrrolidin-2-one), and 4-guanidinobutanoate, perhaps contributing to GABA-mediated muscle growth in response to exercise [35].

Other metabolic changes associated with high endurance included reduced diacylglycerols (DAGs) and fatty acid (FA)-carnitine and increased acylated carnitine. Alterations in these lipids may suggest enhanced hydrolysis of DAGs, shuttling of FA intracellularly, followed by fatty acid oxidation and energy generation [36]. Fatty acids and lipids are preferred substrates for exercising the muscle, and the emerging data suggest a greater beta oxidation of fatty acids in athletes belonging to higher endurance sports. Hence, those athletes are perhaps more capable of activating lipolysis during physical activity than moderate-endurance athletes. Furthermore, accumulation of acylated carnitine may provide a favorable effect on the recovery from exercise stress since carnitine can reduce post-exercise plasma lactate and prevent cellular damage [37]. Citrate and isocitrate were also significantly increased in high-endurance elite athletes, indicating enhanced aerobic energy generation through TCA.

### Metabolites associated with power athletes

Changes in creatine, creatinine, and guanidinoacetate were significant between high- and moderate-power athletes. Whereas creatine increased in the high-power group, its breakdown product (creatinine) and precursor (guanidinoacetate) were both significantly reduced, thus maintaining the previously reported balance of creatine metabolism [38]. Creatine (Cr) and creatine phosphate (CrP) play essential roles in the storage and transmission of phosphate-bound energy. Changes in creatine homeostasis in high-power athletes may suggest more adaptable muscular storage of CrP that during exercise can constitute an essential source for high energy to replenish ATP in the first few seconds of intense activity. Other energy-related metabolites elevated in high-power athletes were 3-methylxanthine and 7-methylxanthine (adenine breakdown products), perhaps reflecting heightened utilization of fuel substrates in several metabolic pathways [39]. Xanthine supplementation allows athletes to exercise at a greater power output for longer times [40]. Additionally, N-acetylcarnosine was significantly reduced in high-power athletes. This metabolite acts as oxidative stress scavenger in muscles especially against lipid peroxidation through its imidazolium group that stabilizes adducts formed at the primary amino group [41]. Various derivatives of phosphatidates were increased with increased power, perhaps reflecting changes in cellular membrane dynamics in response to oxidative stress [42]. Among those, inositol phospholipids were previously shown to accumulate in response to muscle contraction during hypoxia [43]. Another metabolite likely to be a result from stress-induced membrane dynamics is 12,13-DHOME. This long-chain fatty acid enhances adipogenesis and inhibits asteogenesis due to its role as a proliferator-activated receptor (PPAR) gamma 2 ligand [44].

### Global stress response in both high-power and high-endurance athletes

Intensive exercise has been implicated in the promotion of free radical generation in active skeletal muscle resulting in the formation of oxidized lipids [42]. Overall in both power and endurance athletes, there was a clear stress metabolic response. Changes in gamma-gultamyl amino acids, associated with elevated cysteine-glutathione disulfide (change 0.24, nominal *p* value of 0.03), between high- and moderate-performance athletes may indicate active gamma-glutamyl cycle that plays an important role in the glutathione-mediated radical detoxification during oxidative stress [45]. The cycle involves synthesis and degradation of glutathione by transferring gamma-glutamyl functional groups from glutathione to an amino acid, leaving the cysteine products intact, which leads to the preservation of intracellular homeostasis in case of oxidative stress [46, 47]. Reduction in serum levels of gamma-glutamyl-amino acids in high-performance athletes (both high power and high endurance) may indicate increased glutathione synthesis. The accumulation of glutathione in the blood stream marks increased oxidative stress and reactive oxygen species scavenging activity.

### Gender-related differences

Despite lack of FDR significant differences in metabolites associated with endurance in males versus females, differences in a number of metabolites were nominally significant, including a number of gamma-glutamyl amino acids and steroid metabolites among others. Differences in these metabolites between high and moderate levels of endurance were mostly going in the same direction in males and females but were more pronounced in females. As per power-associated metabolites, there were FDR significant differences between males and females in a number of metabolites including TCA-mediators such as malate, fumarate, succinate, and alpha keto glutarate as well as lactate where in females there was increase with higher power with no FDR significant effects in males. These gender-related differences need to be further investigated, especially in light of low number of studied females (*n* = 20).

### Study limitations

One main limitation of this study is the relatively low number of participants, especially the females; therefore, a replication study is essential for confirmation of these findings. Furthermore, since athletes’ blood samples were collected at multiple sites, a batch effect was inevitable, likely attenuating correlations between metabolite concentrations and sports class. This batch effect may have included various crucial pre-analytical features that can significantly influence the metabolic profiling of samples such as the blood collection process and time (IN or OUT of competition) and transportation conditions, including time to reach anti-doping laboratories, sample processing, and sample storage [48]. Despite these factors, clear signatures were identified after correcting for potential confounders. Additionally, the lack of information about participants including their age, ethnicity, and body mass index was another major limitation of this study. However, the young age of elite athletes in general and the wide range of sports included in this study may have diluted out other potential confounders. Ambiguity in the exact description of the subcategories of athletes’ sports was an additional issue this study has faced due to the limited information provided by the anti-doping laboratories following the strict anonymization process. This has prompted the adoption of the general sports class grouping based on previously published work [21] despite the differences among different members of the same team such as such as breast-stroke and freestyle swimming or football midfielders and goal keepers. Another limitation of this study is the group number bias as some sports were overrepresented and others underrepresented. Finally, differences in dietary intake between high- and moderate-power and endurance elite athletes, including various supplements, medications, and other ergogenics, may have influenced their metabolic profile [49]. Such differences are difficult to account for as they vary among different sports and athletes and are not usually publicized. Taken all these limitations into account, it is critical to stress that this is a pilot study that needs further replication and validation as finding biomarkers from the identified differentiating significant compounds still requires optimization of target-specific analytical methods and validation of these methods with their reference materials and proficiency tests [50].

## Conclusion

The emerging data provide a comprehensive snapshot of athletes metabolism based on their sports class as well as small molecule markers of fitness, which requires further validation. Metabolomics of elite athletes classified according to their sports class into endurance or power revealed for the first time changes in metabolites reflecting sex steroid hormones biosynthesis and oxidative stress substrates (glutathione metabolism). The analysis confirmed previously reported changes in the consumption of energy substrates in glycolysis [51], lipolysis [52, 53], adenine nucleotide catabolism [54], and amino acid catabolism [15] in response to exercise [1, 55, 56]. These metabolic signatures could be utilized as pilot indicators of excessive trainability associated with elite athletic performance with potential applications in directing future training programs, preventing potential disorders associated with excessive exercise as well as improving their overall performance. Changes in these metabolic signatures may also provide valuable clues for anti-doping research related to Athlete Biological Passport.

## Additional files

Additional file 1: Materials and Methods. (DOCX 16 kb)

Additional file 2: Table S1. Comparison of previously published metabolite changes in plasma at 60 min after completion of exercise [1] and their corresponding PC2 loading values obtained in this study. **Table S2.** Metabolites differentiating between moderate- and high-endurance athletes (*p* ≤ 0.05). **Table S3.** Metabolites differentiating between moderate- and high-endurance athletes (*p* ≤ 0.05) in males only. **Table S4.** Pearson’s Correlations between various sex steroid metabolites. Significant *p* values are highlighted (* < 0.05, ** < 0.01, *** < 0.001). **Table S5.** Metabolites differentiating between moderate- and high-power athletes (*p* ≤ 0.05). **Table S6.** Metabolites differentiating between moderate- and high-power athletes (*p* ≤ 0.05) in males only. **Table S7.** Gender-endurance interaction metabolites. Columns A–F show the effect of endurance on gender-interaction metabolites in males only. Columns H to L show the different effect in females. **Table S8.** Gender-power interaction metabolites. Columns A–F show the effect of power on gender-interaction metabolites in males only. Columns H to L show the different effect in females. (XLSX 1377 kb)

Additional file 3: Figure S1. Heatmap (left) and hierarchical clustering (right) of steroid metabolites featured in this study. The significant metabolites from the linear model associated with endurance are highlighted in red (right). (PPTX 73 kb)

## Abbreviations

ATP: Adenosine triphosphate
DAGs: Diacylglycerols
ESI: Electrospray ionization
FA: Fatty acid
HESI-II: Heated electrospray ionization
HILIC: Hydrophilic interaction chromatography
LC-MS: Liquid chromatography-mass spectrometry
MVC: Maximal voluntary contraction percentage
OPLS-DA: Orthogonal partial least square discriminant analysis
PCA: Principle component analysis
RP: Reverse phase
TCA: Tricarboxylic acid
UPLC: Ultra-performance liquid chromatography
VO2: Maximal oxygen uptake percentage
