# Supplementary material for: A pilot study comparing the metabolic profiles of elite-level athletes from different sporting disciplines
Source: Sports Med Open. 2018 Jan 5;4:2. doi: 10.1186/s40798-017-0114-z (PMC5756230; doi:10.1186/s40798-017-0114-z)
Supplement: Supplementary file 1 — Materials and Methods. (DOCX 16 kb) [file 40798_2017_114_MOESM1_ESM.docx]

**Materials and Methods**

**Materials**

Authentic standards of d7-glucose, d3-leucine, d8-phenylalanine and d5-tryptophan were purchased from Cambridge Isotope Laboratories (Andover, MA).  D5-hippuric acid, d5-indole acetic acid and d9-progesterone were procured from C/D/N Isotopes, Inc. (Pointe-Claire, Quebec).  Bromophenylalanine was provided by Sigma-Aldrich Co. LLC. (St. Louis, MO) and amitriptyline was from MP Biomedicals, LLC. (Aurora, OH).  Recovery standards of DL-2-fluorophenylglycine and DL-4-chlorophenylalanine were from Aldrich Chemical Co. (Milwaukee, WI). Tridecanoic acid was purchased from Sigma-Aldrich (St. Louis, MO) and d6-cholesterol was from Cambridge Isotope Laboratories (Andover, MA).  Standards for the HILIC dilution series of alpha-ketoglutarate, ATP, malic acid, NADH and oxaloacetic acid were purchased from Sigma-Aldrich Co. LLC. (St. Louis, MO) while succinic acid, pyruvic acid and NAD+ were purchased from MP Biomedicals, LLC. (Santa Ana, CA).

**Methods**

**Sample Preparation:**Following receipt, samples were inventoried and immediately stored at -80^o^C until processed. Samples were prepared using the automated MicroLab STAR® system from Hamilton Company.  Several recovery standards were added prior to the first step in the extraction process for QC purposes.  To remove protein, dissociate small molecules bound to protein or trapped in the precipitated protein matrix, and to recover chemically diverse metabolites, proteins were precipitated with methanol under vigorous shaking for 2 min (Glen Mills GenoGrinder 2000) followed by centrifugation.  The resulting extract was divided into five fractions: two for analysis by two separate reverse phase (RP)/UPLC-MS/MS methods with positive ion mode electrospray ionization (ESI), one for analysis by RP/UPLC-MS/MS with negative ion mode ESI, one for analysis by HILIC/UPLC-MS/MS with negative ion mode ESI, and one sample was reserved for backup. Samples were placed briefly on a TurboVap® (Zymark) to remove the organic solvent.  The sample extracts were stored overnight under nitrogen before preparation for analysis.

**Quality Control (QC):**  Several types of controls were analyzed in concert with the experimental samples: a pooled matrix sample generated by taking a small volume of each experimental sample (or alternatively, use of a pool of well-characterized human plasma) served as a technical replicate throughout the data set; extracted water samples served as process blanks; and a cocktail of QC standards that were carefully chosen not to interfere with the measurement of endogenous compounds were spiked into every analyzed sample, allowed instrument performance monitoring and aided chromatographic alignment.  Instrument variability was determined by calculating the median relative standard deviation (RSD) for the standards that were added to each sample prior to injection into the mass spectrometers.  Overall process variability was determined by calculating the median RSD for all endogenous metabolites (i.e., non-instrument standards) present in 100% of the pooled matrix samples.  Experimental samples were randomized across the platform run with QC samples spaced evenly among the injections.

**Ultrahigh Performance Liquid Chromatography-Tandem Mass Spectroscopy (UPLC-MS/MS):**  All methods utilized a Waters ACQUITY ultra-performance liquid chromatography (UPLC) and a Thermo Scientific Q-Exactive high resolution/accurate mass spectrometer interfaced with a heated electrospray ionization (HESI-II) source and Orbitrap mass analyzer operated at 35,000 mass resolution.  The sample extract was dried then reconstituted in solvents compatible to each of the four methods. Each reconstitution solvent contained a series of standards at fixed concentrations to ensure injection and chromatographic consistency.  One aliquot was analyzed using acidic positive ion conditions, chromatographically optimized for more hydrophilic compounds. In this method, the extract was gradient eluted from a C18 column (Waters UPLC BEH C18-2.1x100 mm, 1.7 µm) using water and methanol, containing 0.05% perfluoropentanoic acid (PFPA) and 0.1% formic acid (FA).  Another aliquot was also analyzed using acidic positive ion conditions, however it was chromatographically optimized for more hydrophobic compounds.  In this method, the extract was gradient eluted from the same afore mentioned C18 column using methanol, acetonitrile, water, 0.05% PFPA and 0.01% FA and was operated at an overall higher organic content.  Another aliquot was analyzed using basic negative ion optimized conditions using a separate dedicated C18 column.  The basic extracts were gradient eluted from the column using methanol and water, however with 6.5mM Ammonium Bicarbonate at pH 8. The fourth aliquot was analyzed via negative ionization following elution from a HILIC column (Waters UPLC BEH Amide 2.1x150 mm, 1.7 µm) using a gradient consisting of water and acetonitrile with 10mM Ammonium Formate, pH 10.8. The MS analysis alternated between MS and data-dependent MS/MS scans using dynamic exclusion.  The scan range varied slighted between methods but covered 70-1000 m/z.  Raw data files are archived and extracted as described below.

**Data Extraction and Compound Identification:** Raw data was extracted, peak-identified and QC processed using Metabolon’s hardware and software.  These systems are built on a web-service platform utilizing Microsoft’s. NET technologies, which run on high-performance application servers and fiber-channel storage arrays in clusters to provide active failover and load-balancing.  Compounds were identified by comparison to library entries of purified standards or recurrent unknown entities.  Metabolon maintains a library based on authenticated standards that contains the retention time/index (RI), mass to charge ratio (*m/z)*, and chromatographic data (including MS/MS spectral data) on all molecules present in the library.  Furthermore, biochemical identifications are based on three criteria: the correct retention time/index to the authentic standard, the correct m/z within 10ppm of the authentic standard and the correct fragmentation spectrum (MS/MS) to the standard.  The MS/MS scores are based on a comparison of the ions present in the experimental spectrum to the ions present in the library spectrum.  While there may be similarities between these molecules based on one of these factors, the use of all three data points can be utilized to distinguish and differentiate biochemicals.  More than 3300 commercially available purified standard compounds have been acquired and registered into Laboratory Information Management System (LIMS) for analysis on all platforms for determination of their analytical characteristics.  Metabolon data analysts use proprietary visualization and interpretation software to confirm the consistency of peak identification among the various samples.  Library matches for each compound were checked for each sample and corrected if necessary.
