# Supplementary material for: A pilot study comparing the metabolic profiles of elite-level athletes from different sporting disciplines
Source: Sports Med Open. 2018 Jan 5;4:2. doi: 10.1186/s40798-017-0114-z (PMC5756230; doi:10.1186/s40798-017-0114-z)
Supplement: Supplementary file 3 — Heatmap (left) and hierarchical clustering (right) of steroid metabolites featured in this study. The significant metabolites from the linear model associated with endurance are highlighted in red (right). (PPTX 73 kb) [file 40798_2017_114_MOESM3_ESM.pptx]

## Slide 1
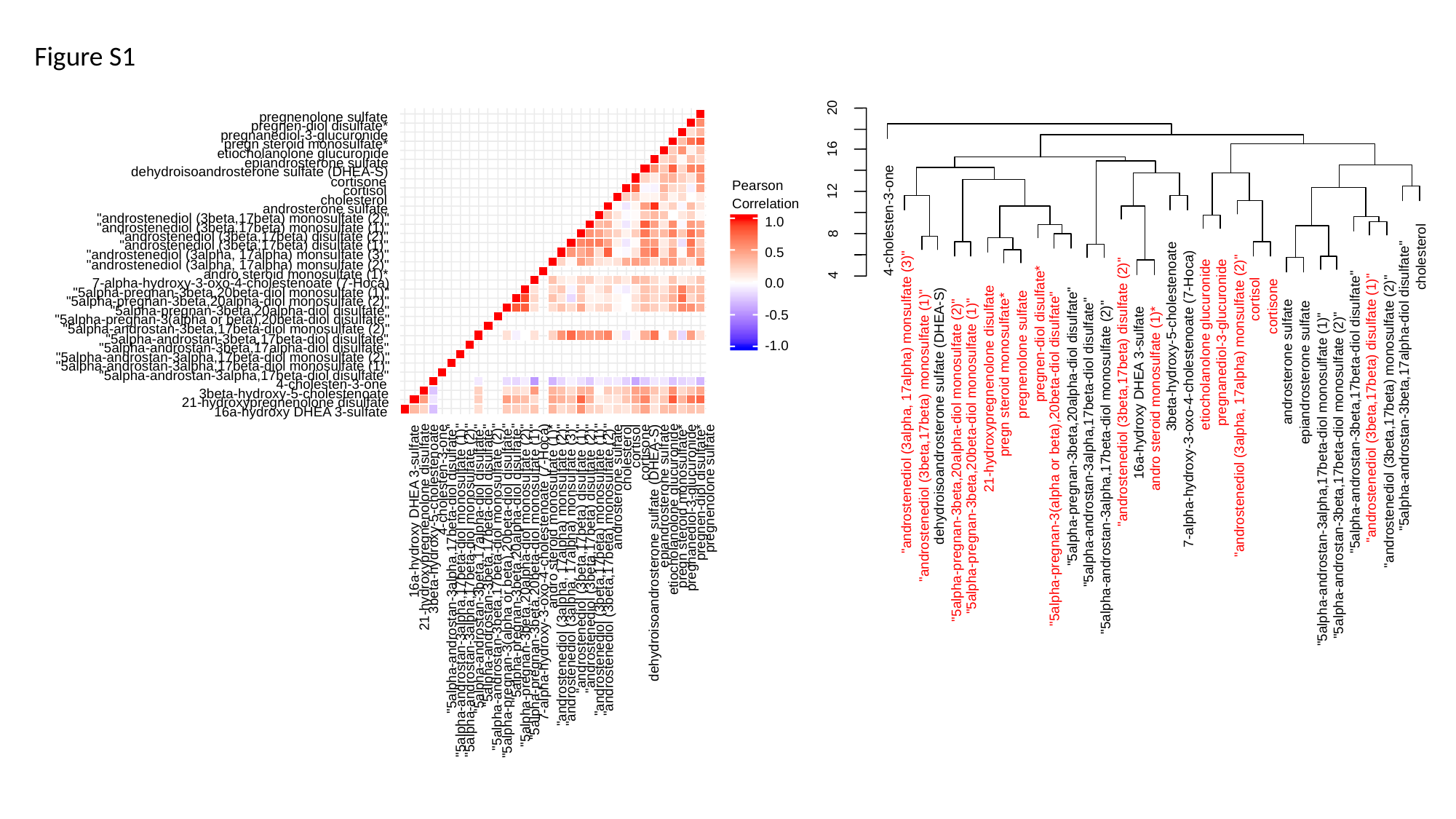

Figure S1
pregnenolone sulfate
"androstenediol (3alpha, 17alpha) monsulfate (3)"
"androstenediol (3alpha, 17alpha) monsulfate (2)"
andro steroid monosulfate (1)*
7-alpha-hydroxy-3-oxo-4-cholestenoate (7-Hoca)
"5alpha-pregnan-3beta,20beta-diol monosulfate (1)"
"5alpha-pregnan-3beta,20alpha-diol monosulfate (2)"
"5alpha-pregnan-3beta,20alpha-diol disulfate"
"5alpha-pregnan-3(alpha or beta),20beta-diol disulfate"
"5alpha-androstan-3beta,17beta-diol monosulfate (2)"
"5alpha-androstan-3beta,17beta-diol disulfate"
"5alpha-androstan-3beta,17alpha-diol disulfate"
"5alpha-androstan-3alpha,17beta-diol monosulfate (2)"
"5alpha-androstan-3alpha,17beta-diol monosulfate (1)"
"5alpha-androstan-3alpha,17beta-diol disulfate"
4-cholesten-3-one
3beta-hydroxy-5-cholestenoate
21-hydroxypregnenolone disulfate
16a-hydroxy DHEA 3-sulfate
pregnen-diol disulfate*
pregnanediol-3-glucuronide
pregn steroid monosulfate*
etiocholanolone glucuronide
epiandrosterone sulfate
dehydroisoandrosterone sulfate (DHEA-S)
cortisone
Pearson
cortisol
cholesterol
Correlation
androsterone sulfate
"androstenediol (3beta,17beta) monosulfate (2)"
1.0
"androstenediol (3beta,17beta) monosulfate (1)"
"androstenediol (3beta,17beta) disulfate (2)"
"androstenediol (3beta,17beta) disulfate (1)"
0.5
0.0
-0.5
-1.0
cortisol
cortisone
cholesterol
4-cholesten-3-one
androsterone sulfate
pregnenolone sulfate
pregnen-diol disulfate*
epiandrosterone sulfate
pregn steroid monosulfate*
pregnanediol-3-glucuronide
etiocholanolone glucuronide
16a-hydroxy DHEA 3-sulfate
andro steroid monosulfate (1)*
3beta-hydroxy-5-cholestenoate
21-hydroxypregnenolone disulfate
dehydroisoandrosterone sulfate (DHEA-S)
"androstenediol (3beta,17beta) disulfate (1)"
"androstenediol (3beta,17beta) disulfate (2)"
"5alpha-pregnan-3beta,20alpha-diol disulfate"
"5alpha-androstan-3beta,17beta-diol disulfate"
"5alpha-androstan-3alpha,17beta-diol disulfate"
"5alpha-androstan-3beta,17alpha-diol disulfate"
"androstenediol (3beta,17beta) monosulfate (1)"
"androstenediol (3beta,17beta) monosulfate (2)"
7-alpha-hydroxy-3-oxo-4-cholestenoate (7-Hoca)
"androstenediol (3alpha, 17alpha) monsulfate (2)"
"androstenediol (3alpha, 17alpha) monsulfate (3)"
"5alpha-pregnan-3beta,20beta-diol monosulfate (1)"
"5alpha-pregnan-3beta,20alpha-diol monosulfate (2)"
"5alpha-androstan-3beta,17beta-diol monosulfate (2)"
"5alpha-androstan-3alpha,17beta-diol monosulfate (1)"
"5alpha-androstan-3alpha,17beta-diol monosulfate (2)"
"5alpha-pregnan-3(alpha or beta),20beta-diol disulfate"
20
16
12
4-cholesten-3-one
8
cholesterol
4
cortisol
cortisone
pregnen-diol disulfate*
3beta-hydroxy-5-cholestenoate
pregnanediol-3-glucuronide
etiocholanolone glucuronide
pregnenolone sulfate
androsterone sulfate
epiandrosterone sulfate
pregn steroid monosulfate*
"5alpha-androstan-3beta,17alpha-diol disulfate"
21-hydroxypregnenolone disulfate
"androstenediol (3beta,17beta) disulfate (2)"
16a-hydroxy DHEA 3-sulfate
7-alpha-hydroxy-3-oxo-4-cholestenoate (7-Hoca)
andro steroid monosulfate (1)*
"androstenediol (3alpha, 17alpha) monsulfate (3)"
"androstenediol (3alpha, 17alpha) monsulfate (2)"
"androstenediol (3beta,17beta) disulfate (1)"
"5alpha-androstan-3beta,17beta-diol disulfate"
dehydroisoandrosterone sulfate (DHEA-S)
"androstenediol (3beta,17beta) monosulfate (2)"
"5alpha-pregnan-3beta,20alpha-diol disulfate"
"androstenediol (3beta,17beta) monosulfate (1)"
"5alpha-androstan-3alpha,17beta-diol disulfate"
"5alpha-pregnan-3beta,20beta-diol monosulfate (1)"
"5alpha-pregnan-3(alpha or beta),20beta-diol disulfate"
"5alpha-pregnan-3beta,20alpha-diol monosulfate (2)"
"5alpha-androstan-3alpha,17beta-diol monosulfate (2)"
"5alpha-androstan-3beta,17beta-diol monosulfate (2)"
"5alpha-androstan-3alpha,17beta-diol monosulfate (1)"
